# Pollen Preference for *Psychotria sp*. is Not Learned in the Passion Flower Butterfly, *Heliconius erato*

**DOI:** 10.1673/031.011.0125

**Published:** 2011-03-03

**Authors:** Christian Salcedo

**Affiliations:** ^1^ Department of Entomology and Nematology, University of Florida, Gainesville, FL 32611; ^2^ McGuire Center for Lepidoptera and Biodiversity, Florida Museum of Natural History Gainesville, FL 32611 -2710, USA

**Keywords:** aggregations, behavior, pollen feeding

## Abstract

*Heliconius* butterflies are known to maximize fitness by feeding on pollen from *Gurania* sp. and *Psiguria* sp. (Cucurbitales: Curcurbitaceae), and *Psychotria* sp. (Gentianales: Rubiaceae). This specialization involves specific physical, physiological, and behavioral adaptations including efficient search strategies in the forest to locate pollen host plants, pollen removal, and pollen external digestion. Reducing pollen host plant search time is crucial to out-compete other flower visitors and to reduce exposure to predators. One way in which this can be achieved is by using chemical cues to learn from experienced foragers in roosting aggregations. Similar strategies have been documented in bumblebees, where inexperienced individuals learn floral odors from experienced foragers. Behavioral experiments using plants preferred by *Heliconius erato* suggest that pollen preference in *H. erato* is an innate trait and consequently learning of chemical cues at roosting aggregations is unlikely.

## Introduction

*Heliconius* butterflies are known to rely significantly on their natural pollen host plants in order to acquire essential amino acids that improve life span and egg production (Brown et al. 1991; Dunlap-Pianka et al. 1977). Preferred pollen host plants include several species from the genera *Gurania* sp. and *Psiguria* sp. (Cucurbitaceae) and several species from the genus *Psychotria* sp. (Rubiaceae). These plants have inflorescences that bear bright orange and red colors, which are important long range cues used by *Heliconius* adults to locate the plants when they are navigating the forest when foraging. Location of these plants has been suggested to be tightly linked with the home range of *Heliconius* populations (Gilbert 1991). By dwelling close to their most important nutritional resource adults maximize their fitness. However, inexperienced adults may be at risk when searching these conspicuous plants in the forest by increasing exposure to predators. In addition, *Heliconius* pollen host plants are also pollinated by hummingbirds (Cardoso de Castro and Cardoso Araujo 2004; Murawski and Gilbert 1986; Stone 1996) and with this competition minimizing search time is important. Pollen host plant search time can be decreased in several ways. One of them assumes that the butterflies are born with no specific preference for a particular pollen host plant and hence they need to learn this preference. Recent evidence in bumblebees shows that inexperienced individuals learn floral odors from experienced foragers by associating flower scented nectar, brought to the nest by the experienced foragers, with a specific chemical cue (Molet et al. 2009). *Heliconius* butterflies form nocturnal aggregations (Wallace 1870), where males and females perch gregariously night after night. These aggregations are stable and often are located near pollen host plants (Mallet 1986). New observations have revealed that females arrive to roost sites with loads of pollen (Salcedo 2010). Young inexperienced *Heliconius* butterflies may be learning pollen odors or taste from experienced foragers that arrive to nocturnal roost sites bearing loads of pollen. Alternatively, preference for natural pollen host plants may be innate so learning would not be necessary. To evaluate if pollen preference in *Heliconius erato* L. (Lepidoptera: Nymphalidae) is innate or learned, choice experiments using *Psychotria sp*. pollen and naïve *H. erato* butterflies were done.

## Materials and Methods

*H. erato* butterflies were reared with *Passiflora biflora* as pollen host plant and held in a 2 × 2 × 3 m outdoor cage. The butterflies were never exposed to their preferred pollen host plants (i.e. *Psychotria* sp., *Gurania* sp. or *Psiguria* sp.) and were trained to feed on red colored feeders with sugar water solution. Based on preliminary tests, colored feeders were used because the butterflies need the color cue to be able to recognize the feeders as a foraging source. In each trial one individual was exposed to two feeding choices (% weight): (a) 30% bee collected pollen (Apiarios Malivern, Panama), 10% sugar, 60% water; and (b) 30% *Psychotria* sp. pollen, 10% sugar, 60% water. In real flowers pollen is offered together with a nectar reward (which is rich in sugars), so the sugar-water solution in the experimental choices was meant to play this role. *Psychotria* sp. pollen was collected from local flowers. Each experiment was carried out in a 2 × 2 × 1.5 m outdoor cage. The solutions were placed in identical red colored feeders

**Table 1.**
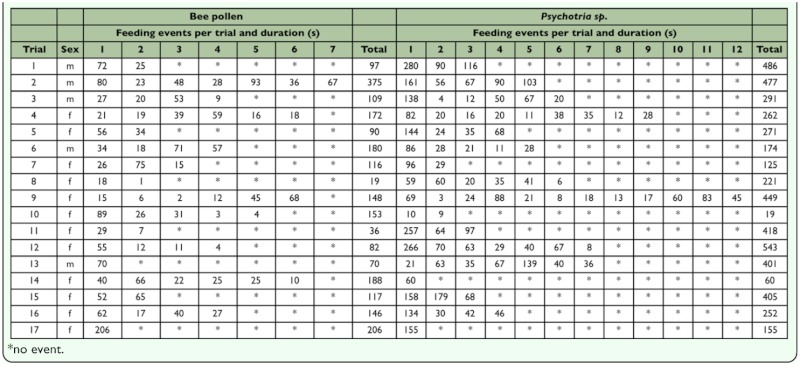
Number and duration of feeding events in pollen-feeding choice tests with naïve *Heliconius erato* butterflies. Feeding choices were artificial red colored feeder with bee collected pollen in water-sugar solution, and artificial red color feeder with manually collected *Psychotria* sp. pollen in sugar-water solution. Each trial lasted 10 min and one individual was used per trial. Whenever an individual started to feed in one of the choices time was recorded with a stopwatch. Total time spent feeding on *Psychotria* sp. pollen is significantly higher compared to time spent in bee pollen (Wilcoxon SR test W = -115 P = 0.0034).

## Results and Discussion

**Figure 1.**
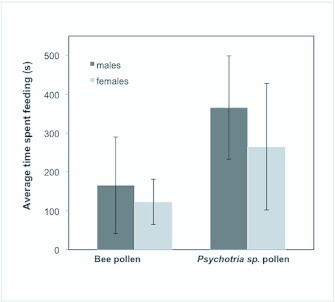
Average feeding time per trial for naïve *Heliconius erato* males and females in pollen-feeding choice experiments. Feeding choices were (A) artificial red colored feeder with bee collected pollen in water-sugar solution, and (B) artificial red color feeder with manually collected *Psychotria* sp. pollen in sugar-water solution. Each trial lasted 10 min and one individual was used per trial. Error bars show standard deviation. High quality figures are available online.

In all trials individual *H. erato* were tested on both choices at least once. The number of feeding events was not significantly different between the choices (Wilcoxon signed rank test: W = -54, P = 0.064), however time spent feeding on the *Psychothria* sp. pollen feeder was significantly higher than time spent feeding on the bee pollen feeder (Wilcoxon SR test: W = -115, P = 0.0034) (Table 1). This suggests that the butterflies naturally prefer to feed on *Psychotria* sp. pollen even having an alternative feeding pollen source available. It is likely then that preference for natural pollen host plants is innate in *H. erato*. Subsequently, learning of pollen preference at roost sites is unlikely. Due to the uneven number of males and females used in the trials (5 males and 12 females) it is difficult to draw conclusions on sex-based preferences. Under natural conditions females tend to forage more because of their physiological and ecological needs in order to increase egg production and have prolonged lifespan (Dunlap-Pianka et al. 1977; O'Brien et al. 2003). The results herein do not follow this trend, overall males spent more time in either of the two choices (Figure 1). It is unknown if *H. erato* are using chemical cues to locate the preferred pollen feeder. Based on field and in-cage observations of feeding behavior, they first use color in the long range (2–10 m) to recognize their potential pollen host plants, then fly towards the flower and hover over before landing to start feeding. Hovering may be used to detect short-range chemical cues. Pollen host plants may have short-range volatile chemical cues that could be produced by the flowers. Recent data demonstrates that *H. erato* is attracted to (*E*)-β-ocimene (unpublished data), a very common semiochemical emitted by flowers to attract pollinators (Knudsen et al. 1993). Pollen grains themselves can also emit volatile chemical cues (Dobson and Bergström 2000). The experimental choices presented here had only a visual cue (red-colored feeders) and two types of pollen, so a plausible explanation for the extended feeding periods on *Psychotria* sp. may be a contact chemical cue (taste) as the major factor in producing the observed results.

The results presented here suggest that once a color cue is used to identify pollen-feeding sources, taste from *Psychotria* sp. pollen grains is enough to assure preference. Nevertheless a combination of flower and pollen—emitted volatile chemical cues is probably necessary in the forest, where chemical noise from numerous other sources is present. Further analyses need to include volatile collection from *Heliconius* pollen host plants, identification of volatiles and evaluation of their role in foraging ecology in cage and field bioassays.
